# Uterine mesenchymal tumours harboring the *KAT6B/A::KANSL1* gene fusion represent a distinct type of uterine sarcoma based on DNA methylation profiles

**DOI:** 10.1007/s00428-024-03935-0

**Published:** 2024-10-11

**Authors:** Felix K. F. Kommoss, Alphonse Charbel, David L. Kolin, Brooke E. Howitt, Martin Köbel, Jen-Chieh Lee, W Glenn McCluggage, Abbas Agaimy, Brendan C. Dickson, Andreas von Deimling, Cheng-Han Lee

**Affiliations:** 1 Institute of Pathology, Heidelberg University Hospital, Heidelberg, Germany; 2 Department of Pathology and Laboratory Medicine, University of British Columbia, Vancouver, BC Canada; 3 Department of Molecular Oncology, British Columbia Cancer Research Institute, Vancouver, BC Canada; 4 Division of Women’s and Perinatal Pathology, Department of Pathology, Brigham and Women’s Hospital, Boston, MA USA; 5 Department of Pathology, Stanford University School of Medicine, Stanford, CA USA; 6 Department of Laboratory Medicine and Pathology, University of Calgary, Calgary, AB Canada; 7 Department and Graduate Institute of Pathology, National Taiwan University Hospital, National Taiwan University College of Medicine, Taipei, Taiwan; 8 Department of Pathology, Belfast Health and Social Care Trust, Belfast, UK; 9 Department of Pathology, Friedrich-Alexander-Universität Erlangen-Nürnberg (FAU), Erlangen, Germany; 10 Department of Pathology and Laboratory Medicine, Sinai Health System and University of Toronto, Toronto, ON Canada; 11 Department of Neuropathology, Heidelberg University Hospital, Heidelberg, Germany; 12 Clinical Cooperation Unit Neuropathology, DKFZ, Heidelberg, Germany; 13 Department of Laboratory Medicine and Pathology, University of Alberta, Edmonton, AB Canada

**Keywords:** KAT6B/A, KANSL1, Uterine sarcoma, DNA methylation

## Abstract

Uterine mesenchymal tumours harboring *KAT6B/A::KANSL1* gene fusions typically exhibit histological and immunophenotypic overlap with endometrial stromal and smooth muscle tumours. To date it remains uncertain whether such neoplasms should be regarded as variants of smooth muscle or endometrial stromal neoplasm, or if they constitute a distinct tumour type. In this study we investigated DNA methylation patterns and copy number variations (CNVs) in a series of uterine tumours harboring *KAT6B/A::KANSL1* gene fusions in comparison to other mesenchymal neoplasms of the gynecological tract. Unsupervised hierarchical clustering and t-SNE analysis of DNA methylation data (Illumina EPIC array) identified a distinct cluster for 8/13 *KAT6B/A::KANSL1* tumours (herein referred to as core cluster). The other 5 tumours (herein referred to as outliers) did not assign to the core cluster but clustered near various other tumour types. CNV analysis did not identify significant alterations in the core cluster. In contrast, various alterations, including deletions at the *CDKN2A/B* and *NF1* loci were identified in the outlier group. Analysis of the DNA methylation clusters in relation to histological features revealed that while tumours in the core *KAT6B/A::KANSL1* cluster were histologically bland, outlier tumours frequently exhibited “high-grade” histologic features in the form of significant nuclear atypia, increased mitotic activity and necrosis. Three of the five patients with outlier tumours died from their disease while clinical follow-up in the remaining two patients was limited (less than 12 months). In comparison, none of the 7 out of 8 patients with tumors in the core *KAT6B/A::KANSL1* sarcoma cluster, where follow-up was available, died from disease. Furthermore, only 1 out of 7 patients recurred (mean follow-up of 30 months). In conclusion, *KAT6B/A::KANSL1* uterine sarcoma is a molecularly unique type of uterine tumour that should be recognized as a distinct entity. These tumors typically exhibit low-grade histologic features but are occasionally morphologically high-grade; the latter have a DNA methylation profile different from the typical low-grade neoplasms and may be associated with aggressive behaviour.

**Supplementary Information:**

The online version contains supplementary material available at 10.1007/s00428-024-03935-0.

## Introduction

Uterine mesenchymal tumour harboring t(10;17)(q22;q23) were first reported in 2004 as cellular leiomyomas with the chromosome 10 breakpoint mapped to the gene *KAT6B* (formerly known as MORF) [1]. In 2015, RNA-sequencing performed on a t(10;17)(q22;q21) retroperitoneal uterine-type leiomyoma with prominent fibrous matrix demonstrated a *KAT6B::KANSL1* gene fusion, which results in a putative in-frame chimeric protein encoded by exons 1 to 3 of *KAT6B* and exons 11 to 15 of *KANSL1* [2]. This was followed by case reports describing a rapidly enlarging *KAT6B::KANSL1* uterine leiomyoma in a postmenopausal woman and a uterine leiomyosarcoma harboring a *KAT6B::KANSL1* gene fusion [3, 4].

Subsequently, there have been two studies describing the clinical and pathologic features of a series of *KAT6B/A::KANSL1* uterine tumours, challenging the classification of these tumours as smooth muscle neoplasms. The first series included 11 *KAT6B::KANSL1* and 2 *KAT6A::KANSL1* tumours [5]. The original histologic diagnoses prior to molecular testing were endometrial stromal sarcoma in 9 cases (7 low-grade, 1 low-grade with progression to high-grade, and 1 uncertain grade), smooth muscle tumour in 2 cases (1 leiomyoma and 1 leiomyosarcoma), uterine tumour resembling ovarian sex cord tumour (UTROSCT) in 1 case and undifferentiated uterine sarcoma in 1 case. Upon central review, most of the tumours were found to have low-grade endometrial stromal sarcoma (LGESS)-like morphology but with “atypical” features. The second series included 12 *KAT6B/A::KANSL1* uterine tumours (including 12 primary and 4 recurrent tumours) [6]. The original histologic diagnoses of the primary neoplasms were smooth muscle tumours in 7 cases (6 leiomyomas and 1 intravascular leiomyomatosis) and endometrial stromal tumours in 5 cases (4 endometrial stromal nodules [ESN] and 1 LGESS), although the diagnoses in two of the recurrent tumours initially diagnosed as leiomyomas were amended to LGESS. There was notable inter-tumoral (primary and recurrent tumour) and intra-tumoral heterogeneity with endometrial stromal tumour-like and smooth muscle tumour-like features present in several cases. RNA-sequencing-based gene expression profiling analysis showed a greater overlap of *KAT6B::KANSL1* tumours with ESN and LGESS compared to leiomyomata and leiomyosarcoma. However, distinct differences in signaling pathways were noted between *KAT6B::KANSL1* tumours and ESN/LGESS. Array comparative genomic hybridization did not identify significant amounts of copy number variations (CNV) in 10 tumors studied, and no pathogenic variants in selected genes were identified in their cDNA data.

Overall, studies to date have shown that *KAT6B/A::KANSL1* uterine tumours can exhibit histologic and immunophenotypic features of both low-grade endometrial stromal and smooth muscle neoplasms [5, 6]. Moreover, *KAT6B/A::KANSL1* uterine tumours have demonstrated malignant potential with a risk for metastasis/recurrence despite typically bland cytomorphology and often a circumscribed tumour border. The current uncertainty regarding the precise histotype categorization and clinical behaviour is troubling for pathologists; in particular, *KAT6B/A::KANSL1* tumours challenge the diagnostic dogma in both uterine stromal and smooth muscle classification schema. For instance, if *KAT6B/A::KANSL1* uterine tumours were to be considered a form of low-grade endometrial stromal neoplasm, the typically circumscribed tumour border would challenge our current diagnostic criteria that separate LGESS from ESN. Alternatively, if they were to be classified as smooth muscle tumours, the typically bland cytomorphology and low mitotic rate make it challenging to recognize its malignant potential based on conventional diagnostic criteria for malignancy in uterine smooth muscle tumours. As such, it is important to ascertain the nature and the precise classification of *KAT6B/A::KANSL1* uterine tumours.

DNA methylation profiling has recently shown great promise in providing insights into the classification of central nervous system (CNS) neoplasms and mesenchymal tumours, including gynecologic tract mesenchymal tumours such as LGESS, high-grade endometrial stromal sarcoma (HGESS) and SMARCA4-deficient uterine sarcoma (SDUS) [7–10]. In this study, we employed global DNA methylation profiling and CNV analyses to gain insights into the classification of *KAT6B/A::KANSL1* uterine tumour in comparison to other uterine mesenchymal tumours.

## Material and methods

### Study cohort

We collected a multicenter cohort of uterine tumours harbouring *KAT6B/A::KANSL1* gene fusions. Cases were identified through review of pathology archives to which the authors are affiliated with, as well as the consultation files of the authors. All the tumours had previously been analysed by next generation sequencing at the submitting institutions as previously described [5] (synthesis of cDNA and downstream Illumina Truesight RNA fusion panel or TruSeq Exome sequencing followed by RT-PCR). For all cases, haematoxylin and eosin (H&E) stained slides from formalin-fixed paraffin embedded (FFPE) tissue blocks were available. Medical records were reviewed for clinical and follow-up data.

### DNA extraction and array-based analysis

DNA was extracted from macrodissected FFPE tumour tissue using the Maxwell® 16 FFPE Plus LEV DNA Kit or the Maxwell® 16 Tissue DNA Purification Kit (for frozen tissue) on the automated Maxwell device (Promega, Madison, WI, USA) according to the manufacturer’s instructions. A minimum of 100 ng DNA was subjected to bisulfite conversion and processed on the Illumina Infinium EPIC (850 k) BeadChip (Illumina, San Diego, USA) according to the manufacturer’s instructions.

### DNA methylation analysis

DNA methylation data analysis was performed in R using packages from Bioconductor [11]. Data were normalized by using background correction and dye bias correction (shifting of negative control probe mean intensity to zero and scaling of normalization control probe mean intensity to 20,000). Probes targeting sex chromosomes, probes containing multiple single nucleotide polymorphisms and probes that could not be uniquely mapped were removed. For subsequent DNA methylation analyses, we included a previously compiled methylation dataset of a large cohort of various uterine sarcomas with different gene fusions including LGESS (*n* = 18) with *JAZF1::SUZ12*, *JAZF1::PHF1*, *EPC1::PHF1* and *MEAF6::PHF1* gene fusions, HGESS (*n* = 31) with *YWHAE::NUTM2A/B*, *ZC3H7B::BCOR*, *BCORL1*-rearrangements, and *BCOR* ITD, as well as other uterine mesenchymal neoplasms such as UTROSCT (*n* = 23), leiomyoma (*n* = 27), leiomyosarcoma (*n* = 37), *DICER1*-mutant embryonal rhabdomyosarcoma (ERMS; *n* = 22) and SDUS (*n* = 6) [7, 9, 10, 12]. For unsupervised 2D representation of pairwise sample correlations, dimensionality reduction by t-distributed stochastic neighbor embedding (t-SNE) was performed using the 10,000 most variable probes, a perplexity of 10 and 3,000 iterations. The stability of methylation groups was assessed by altering the number of the most variable probes.

### Copy number analysis

CNV analysis was performed by analysing methylation array data using the R-package *copynumber* [13]. Gene amplifications or deletions were detected by manual inspection. The Genomic Index (GI) was calculated as previously described (total number of segmental gains or losses^2^/number of involved chromosomes) [14]. The upper and lower thresholds for segmental gains and losses were set at 0.1 and -0.1 (log2), respectively.

### Meta-analysis

We performed a literature review to identify all gynecological and soft-tissue tumours with *KAT6B/A::KANSL1* gene fusion previously reported. We conducted a systematic search in most appropriate publications and in the electronic database PubMed using a combination of keywords. Furthermore, we reviewed the reference lists of identified articles manually.

## Results

### Clinicopathologic characteristics of KAT6B/A::KANSL1 uterine sarcoma

Our study cohort consisted of 16 tumours harboring a *KAT6B/A::KANSL1* gene fusion (*KAT6B::KANSL1*, *n* = 15; *KAT6A::KANSL1*, *n* = 1). Median age at diagnosis was 50.5 years (mean age 53 years, range 32 – 83 years). Clinicopathological information on 11 of the 16 cases studied has previously been published [5]. Clinicopathological and molecular characteristics of the entire current study cohort are summarized in Table 1.

**Table 1:** Clinicopathological and molecular characteristics of the study cohort (*n* = 16). Endometrial stromal tumour (EST), highly-cellular leiomyoma (HCL), haemangiopericytoma-like (HPC-like). *sheet-like growth of epithelioid/round cells with monomorphic nuclear atypia, increased mitoses, pericellular hyalinization and multifocal necrosis

Case ID	Age	Alteration	Fusion detection method	Histology	Peripheral border	Sex cord-like changes	Necrosis	Mitoses/10 Hpf	Methylation class	GI	Deep deletion
1	47	*KAT6B::KANSL1*	Illumina TruSight RNA Fusion Panel	EST vs HCL	Well-circumscribed	Present	Present	22	core *KAT6B/A::KANSL1*	3	
2	50	*KAT6B::KANSL1*	Illumina TruSight RNA Fusion Panel	EST (fibrous)	Well-circumscribed	Present	Absent	1	core *KAT6B/A::KANSL1*	3	
3	32	*KAT6B::KANSL1*	Illumina TruSight RNA Fusion Panel	EST vs HCL	Infiltrative	Absent	Present	2	core *KAT6B/A::KANSL1*	9	
4	46	*KAT6B::KANSL1*	Illumina TruSight RNA Fusion Panel	EST	Infiltrative	Present	Absent	12	core *KAT6B/A::KANSL1*	11	
5	51	*KAT6B::KANSL1*	Illumina TruSight RNA Fusion Panel	EST (prominent HPC-like vasculature)	NA	Absent	Absent	3	core *KAT6B/A::KANSL1*	24	
6	81	*KAT6B::KANSL1*	Illumina TruSight RNA Fusion Panel	EST (prominent HPC-like vasculature)	Well-circumscribed	Absent	Present	1	core *KAT6B/A::KANSL1*	33	
7	40	*KAT6B::KANSL1*	Illumina TruSight RNA Fusion Panel	EST vs HCL	Well-circumscribed	Absent	Absent	2	core *KAT6B/A::KANSL1*	65	
8	58	*KAT6B::KANSL1*	Illumina TruSight RNA Fusion Panel	EST (prominent HPC-like vasculature)	Well-circumscribed	Present	Present	3	core *KAT6B/A::KANSL1*	266	
9	34	*KAT6B::KANSL1*	Illumina TruSight RNA Fusion Panel	EST vs HCL	Well-circumscribed	Present	Absent	1	Outlier (LMO/LMS)	8	*NF1*
10	62	*KAT6A::KANSL1*	TruSeq RNA Exome Kit, RT-PCR	Atypical	Well-circumscribed	Present	Present	16	Outlier (HGESS)	152	*CDKN2A/B*
11	50	*KAT6B::KANSL1*	Illumina TruSight RNA Fusion Panel	Atypical	NA	Absent	Present	> 20	Outlier (HGESS)	272	
12	49	*KAT6B::KANSL1*	TruSeq RNA Exome Kit	EST (fibromyxoid)	Well-circumscribed, focal myometrial entrapment	Present	Present	3	Outlier (ERMS)	300	*CDKN2A/B, NF1*
13	53	*KAT6B::KANSL1*	Illumina TruSight RNA Fusion Panel	Atypical	Infiltrative	Absent	Present	> 20	Outlier (LMO/LMS)	3796	
14	62	*KAT6B::KANSL1*	Illumina TruSight RNA Fusion Panel	EST	Well-circumscribed	Present	Present	3	NA	NA	
15	54	*KAT6B::KANSL1*	Illumina TruSight RNA Fusion Panel	EST (prominent HPC-like vasculature)	Well-circumscribed, focal tongue-like protrusions	Absent	Absent	1	NA	NA	
16	83	*KAT6B::KANSL1*	Illumina TruSight RNA Fusion Panel	EST	Well-circumscribed, focal irregularities	Absent	Present	1	NA	NA	

### DNA methylation profiling of KAT6B/A::KANSL1 uterine sarcoma

**Fig. 1:**
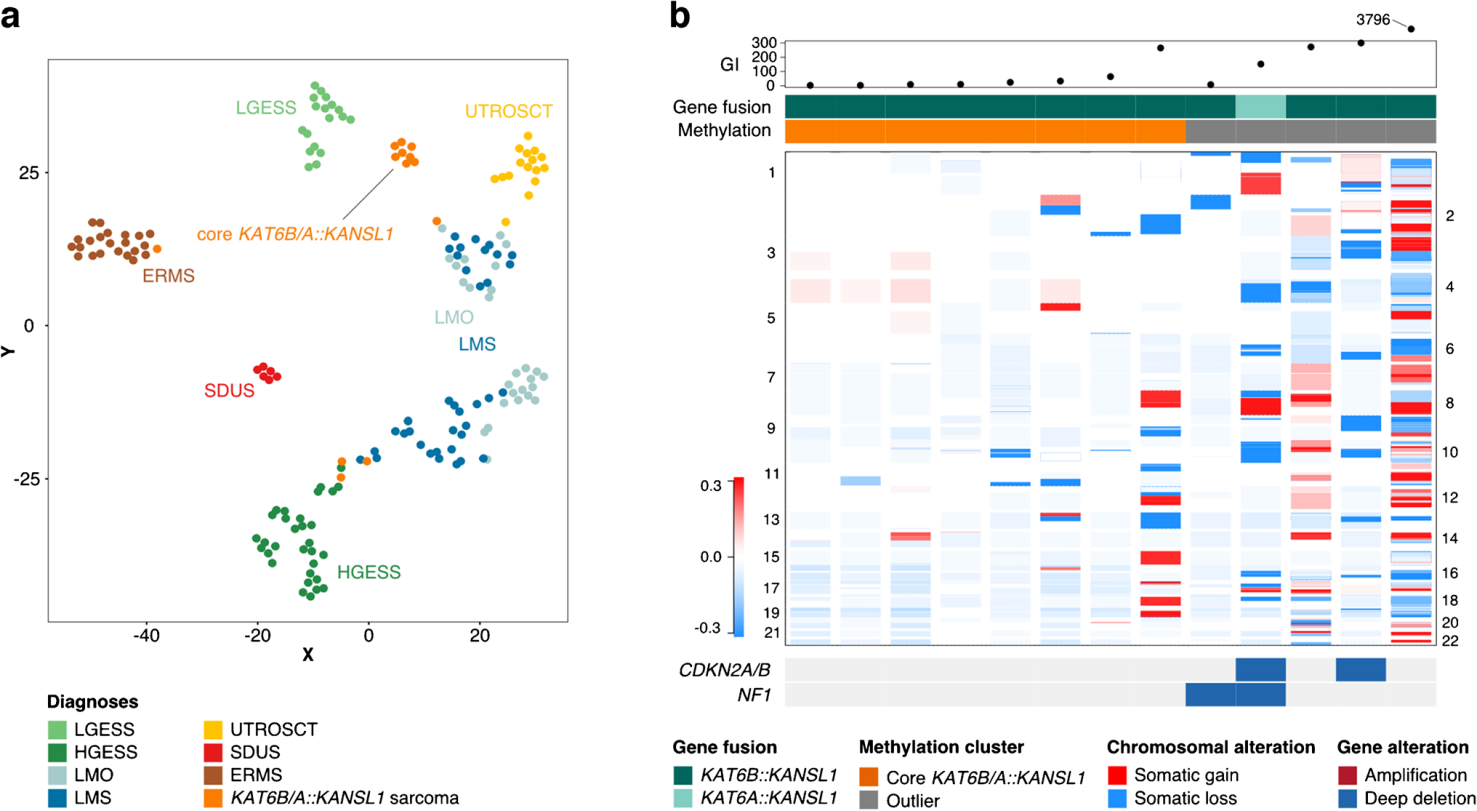
**a** 2D representation of pairwise sample correlation using the 10,000 most variable methylated probes by t-SNE dimensionality reduction. This analysis revealed a distinct “core” cluster of *KAT6B/A::KANSL1* sarcoma (*n* = 8), while 5 tumours with a *KAT6B/A::KANSL1* gene fusion did not cluster with this core group (“outliers”). Samples are coloured according to their institutional diagnoses: Low-grade endometrial stromal sarcoma (LGESS), high-grade endometrial stromal sarcoma (HGESS), leiomyoma (LMO), leiomyosarcoma (LMS), uterine tumour resembling ovarian sex cord tumour (UTROSCT), *SMARCA4*-deficient uterine sarcoma (SDUS), *DICER1*-mutant embryonal rhabdomyosarcoma (ERMS), *KAT6B/A::KANSL1* uterine sarcoma (*KAT6B*/*A*::*KANSL1* sarcoma). **b** Case-by-case copy number profiles of *KAT6B/A::KANSL1* uterine sarcoma with chromosomal gains depicted in red and losses shown in blue. Above Genomic index (total number of segmental gains or losses^2^/number of involved chromosomes) of *KAT6B/A::KANSL1* uterine sarcoma, indicative of genomic complexity (*n* = 13) as well as fusion transcript identified, and DNA methylation class assignment are annotated as indicated by the figure's legend

FFPE material for DNA extraction and subsequent array-based analysis was available for 13 of 16 tumours. Genome wide DNA methylation analysis of the study cohort identified a distinct cluster of tumours harbouring a *KAT6B::KANSL1* gene fusion (“core *KAT6B/A::KANSL1* cluster”), including 8 of 13 tumours analysed herein (Fig. 1a). This cluster was distinct from other clusters of endometrial stromal tumours (LGESS and HGESS), smooth muscle tumours (leiomyoma and leiomyosarcoma), UTROSCT, ERMS and SDUS. Interestingly, 5 tumours harboring a *KAT6B/A::KANSL1* gene fusion, hereafter referred to as “outliers”, did not cluster with the core cluster. Two of the outliers clustered near the HGESS cluster, two were close to the leiomyoma/leiomyosarcoma clusters, and one was adjacent to the ERMS cluster. A review of the immunohistochemical profiles of the 5 outliers revealed that their characteristics did not align with the specific tumor groups which they clustered near (see Supplementary Table 1). Specifically, the two tumors clustering near leiomyoma/leiomyosarcoma were both negative for Desmin and H-Caldesmon. The tumors clustering near HGESS showed profiles atypical for HGESS: one was diffusely positive for Desmin, while the other was negative for Cyclin D1. Additionally, the outlier that clustered with ERMS was negative for Desmin. Furthermore, besides *KAT6B/A::KANSL1* gene fusions, RNA sequencing did not identify prototypical gene fusions typically seen in HGESS or smooth muscle tumors in any of the outlier tumours. Clinically, 3 of the patients with outlier tumours died from progressive disease at 2, 10 and 47 months after the initial diagnosis. Follow-up of the remaining 2 patients was limited (10 and 32 months) and there was no evidence of disease recurrence. In comparison, none of the 7 out of 8 patients with tumors in the core *KAT6B/A::KANSL1* cluster, where follow-up was available, died from disease. Furthermore, only 1 out of 7 patients had a recurrence and is alive with disease, although follow-up was limited, with a mean of 30 months (median 23 months).

### Copy number alterations in KAT6B/A::KANSL1 uterine sarcoma

CNV analysis did not identify significant alterations in the core *KAT6B/A::KANSL1* cluster tumours. In contrast, CNV analysis identified various alterations in the outlier group, including deletions at the *CDKN2A*/*B* and *NF1* loci in one case, a *NF1* deletion in another, and *CDKN2A*/*B* deletions in another (Fig. 1b). The median GI for the core *KAT6B/A::KANSL1* cluster tumours was 17 (mean GI 51.7, range 3—266), in stark contrast to DNA methylation outlier tumours which showed a median GI of 272 (mean GI 906, range 8—3796) (Table 1).

### Pathologic features of “core cluster” and “outlier” sarcoma

**Fig. 2:**
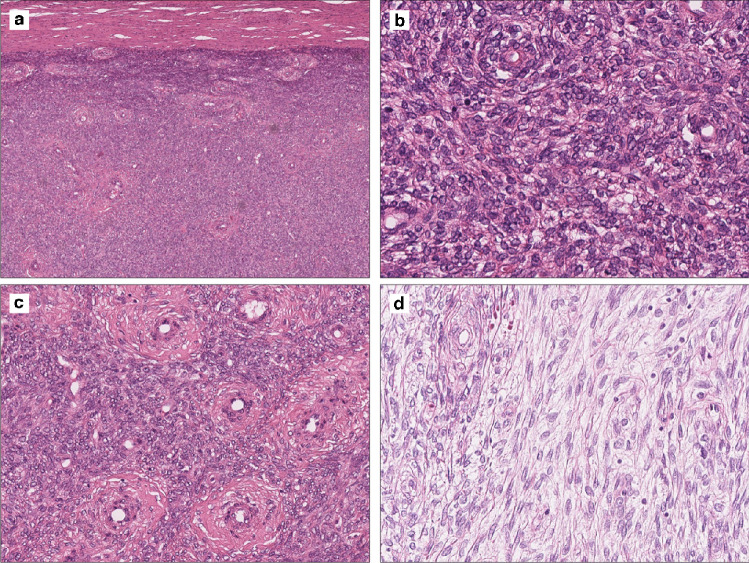
Histological features of *KAT6B::KANSL1* uterine sarcoma assigned to the “core *KAT6B/A::KANSL1* cluster” by DNA methylation profiling (see Fig. 1a). Tumours consisted of often well circumscribed (**a**) cellular proliferations of ovoid cells that mimic a low-grade endometrial stromal tumour (**b**) but contained scattered thick-walled vessels (**c**) or showed a spindle cell pattern (whorled growth pattern) with fibrous to fibromyxoid stroma and bland cytology (**d**)

**Fig. 3:**
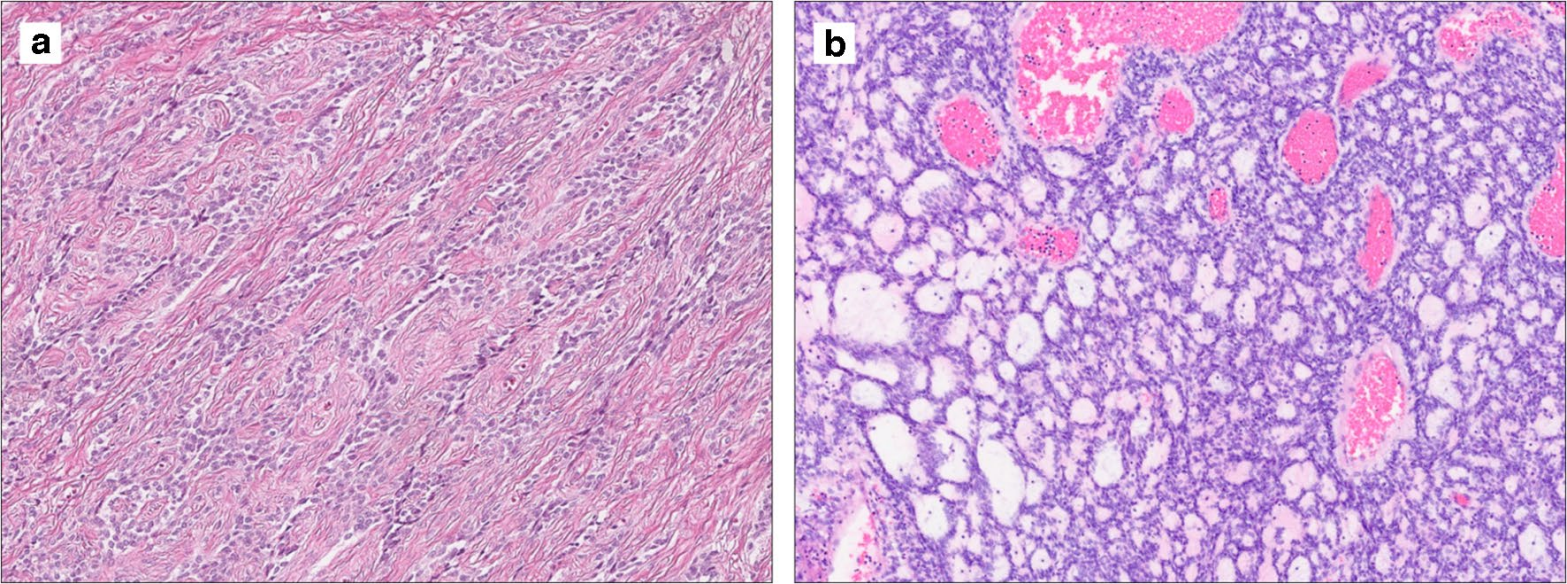
Case 2 assigned to the “core *KAT6B/A::KANSL1* cluster” by DNA methylation profiling shows focal sex cord elements with a nested and trabecular growth pattern (**a**). Case of a *KAT6B::KANSL1* uterine sarcoma (case 14), which was not analysed by array-based methods, displaying focal sex cord elements with a sieve-like to follicular arrangement (**b**)

Histopathological and immunohistochemical features of a subset of cases have previously been described in detail elsewhere [5]. Histological features of all cases are detailed in Table 1. Analysis of DNA methylation cluster assignments in relation to histological features revealed that while tumours in the core *KAT6B/A::KANSL1* cluster were histologically bland, outlier tumours frequently exhibited “high-grade” morphological features. In detail, core *KAT6B/A::KANSL1* cluster tumours consisted of either a diffuse cellular population of ovoid cells that mimicked an endometrial stromal tumour, but contained scattered thick-walled vessels, or showed a spindle cell pattern (whorled growth pattern) with fibrous to fibromyxoid stroma and prominent capillary network (pericytoma-like) with bland cytology (Fig. 2a-d). These tumours exhibited little in the way of nuclear atypia and 4 of 8 of them displayed focal areas with a sex cord-like pattern (Fig. 3a). While most cases showed a well-circumscribed peripheral tumour border, an infiltrative growth pattern was noted in two. Areas of necrosis were present in 4 of 8 tumours and mitotic count ranged from 1 to 22 mitoses per 10 HPF.

**Fig. 4:**
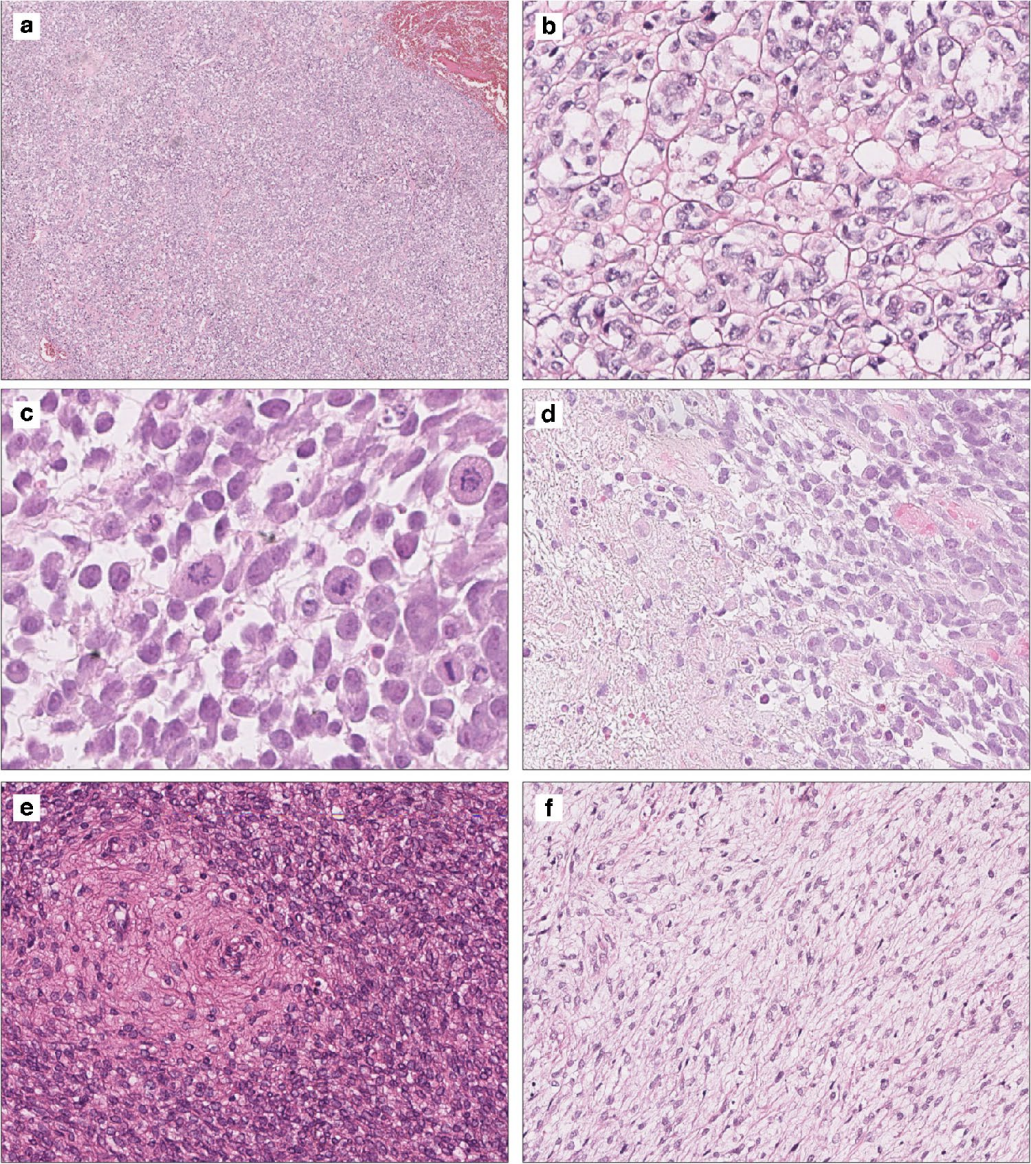
Histological features of *KAT6B/A::KANSL1* uterine sarcoma clustering as “outliers” by DNA methylation profiling (see Fig. 1a). Three of the 5 outliers were highly cellular (**a**) and showed high-grade features with cellular epithelioid round cell histology showing concurrent monomorphic nuclear atypia and increased mitotic index (**b** and **c**) often with tumour cell necrosis (**d**). The remaining 2 outliers (cases 9 and 12) consisted of monomorphic round/ovoid cells (**e**) and fibromyxoid spindle cell proliferations (**f**), respectively

In contrast, 3 of 5 outliers showed uniform high-grade features with a diffuse cellular proliferation of epithelioid round cells exhibiting monomorphic nuclear atypia and readily identifiable mitotic activity (16 to > 20 mitoses per 10 HPF) (Fig. 4a-d). The remaining 2 outliers (cases 9 and 12) consisted of monomorphic round/ovoid cells and a fibromyxoid spindle cell proliferation, respectively (Fig. 4e and f). Focal areas with a sex cord-like pattern were present in 3 of 5 outliers. Only one outlier displayed a clear-cut myoinvasive growth pattern. Areas of necrosis were present in 4 of 5 outliers and mitotic count ranged from 1 to > 20 mitoses per 10 HPF.

Of note, the three cases, which did not undergo DNA methylation profiling, were histologically bland and showed a histology akin to the core *KAT6B/A::KANSL1* cluster tumors. Case 14 showed focal sex cord-like elements with a sieve-like to follicular arrangement (Fig. 3b).

### Meta-analysis of outcomes in KAT6B/A::KANSL1 sarcoma

Twenty-eight tumours harbouring a *KAT6B/A::KANSL1* gene fusion have been reported previously [2–6], and with the addition of cases we report in the current study, the total is 33 (Table 2) (11 of the cases we report have also been previously documented (5)). All tumours except one retroperitoneal tumour were located in the uterus. The median age of patients with *KAT6B/A::KANSL1* tumours was 50 years (mean age 53 years, range: 33 – 83 years). 24 of 28 tumours (86%) were low stage (FIGO uterine sarcoma stage IA or IB) where this information was available. The others were stage IIA/B, IIIB, or IVB (synchronous metastases to the lungs). The tumours recurred in 7 of 25 patients (28%) and 3 of 27 patients (11%) died of disease (at 2, 10 and 47 months). The mean follow-up time was 37 months (median 22 months).

**Table 2:** Meta analysis of clinical features of 33 reported tumours harboring the *KAT6B/A::KANSL1* gene fusion. (* follow up updated since initial publication). Endometrial stromal nodule (ESN), low-grade endometrial stromal sarcoma (LGESS), leiomyoma (LMO), leiomyosarcoma (LMS), uterine tumour resembling ovarian sex cord tumour (UTROSCT); total hysterectomy (TH), bilateral salpingectomy (BS), bilateral salpingo-oophorectomy (BSO); no evidence of disease (NED), alive with disease (AWD), dead of disease (DOD)

Study ID	Study	Year	PMID	Initial diagnosis	Alteration	Site	Age	Stage	Therapy	Recurrence	Status at last follow-up	Follow-up (months)
	Panagopoulos et al	2015	25621995	LMO	*KAT6B::KANSL1*	retroperitoneal	45	NA	Resection	no	NED	50
	Ainsworth et al	2019	31027501	LMO	*KAT6B::KANSL1*	uterine	74	IB	TH-BSO	no	NED	6
	Choi et al	2021	33876771	LMS	*KAT6B::KANSL1*	uterine	69	NA	NA	NA	NA	NA
4	Agaimy et al	2022	35575789	LGESS	*KAT6B::KANSL1*	uterine	46	IA	TH-BSO	no	NED	9
1	Agaimy et al	2022	35575789	LGESS	*KAT6B::KANSL1*	uterine	47	IB	Myomectomy, then TH-BSO	no	NED	23
6	Agaimy et al	2022	35575789	LGESS	*KAT6B::KANSL1*	uterine	81	IB	TH-BSO	no	NED	62*
10	Agaimy et al	2022	35575789	UUS	*KAT6A::KANSL1*	uterine	62	IB	TH-BSO	no	NED	32*
9	Agaimy et al	2022	35575789	LGESS	*KAT6B::KANSL1*	uterine	34	IB	TH	no	NED	10
3	Agaimy et al	2022	35575789	LGESS	*KAT6B::KANSL1*	uterine	33	IIA	Myomectomy, then TAH-BSO	no	NED	4*
7	Agaimy et al	2022	35575,789	LGESS	*KAT6B::KANSL1*	uterine	40	IA	Curettage	NA	NA	NA
13	Agaimy et al	2022	35575789	LMS	*KAT6B::KANSL1*	uterine	53	IVB	TH-BSO	NA	DOD	2
12	Agaimy et al	2022	35575789	LMO	*KAT6B::KANSL1*	uterine	49	IB	TAH-BSO, bilateral pelvic lymphadenectomy, omental biopsy	yes (46 months)	DOD	47
11	Agaimy et al	2022	35575,789	LGESS with progression to HGESS	*KAT6B::KANSL1*	uterine, broad ligament	50	NA	TH-BSO	yes (NA)	DOD	10
	Agaimy et al	2022	35575789	LGESS	*KAT6A::KANSL1*	uterine	63	NA	Curettage	NA	AWD	204
	Agaimy et al	2022	35575789	UTROSCT	*KAT6B::KANSL1*	uterine	39	NA	Myomectomy, TAH-BSO and debulking for recurrence	yes (84 months)	AWD	204
8	Agaimy et al	2022	35575789	LGESS	*KAT6B::KANSL1*	uterine	58	IB	TH	yes (NA)	AWD	60*
	Trecourt et al	2023	37307879	IVLM	*KAT6B::KANSL1*	uterine	60	IA	TH-BS	no	NED	29
	Trecourt et al	2023	37307879	LMO	*KAT6B::KANSL1*	uterine	42	IB	TH-BS	no	NED	22
	Trecourt et al	2023	37307879	ESN	*KAT6B::KANSL1*	uterine	50	IB	TH-BS	no	NED	8
	Trecourt et al	2023	37307879	LMO	*KAT6B::KANSL1*	uterine	47	IB	Myomectomy	no	NED	3
	Trecourt et al	2023	37307879	ESN	*KAT6B::KANSL1*	uterine	49	IB	TH	no	NED	40
	Trecourt et al	2023	37307879	IVLM	*KAT6B::KANSL1*	uterine	47	IB	TH-BS	no	NED	6
	Trecourt et al	2023	37307879	LMO	*KAT6B::KANSL1*	uterine	47	IIB	Intravaginal mass resection, TH-BS	yes (26 months)	NED	28
	Trecourt et al	2023	37307879	LMO	*KAT6B::KANSL1*	uterine	40	IA	Myomectomy, uterine recurrence	yes (8 months)	NED	18
	Trecourt et al	2023	37307879	ESN	*KAT6B::KANSL1*	uterine	43	IA	TH	NA	NA	NA
	Trecourt et al	2023	37307879	ESS	*KAT6B::KANSL1*	uterine	68	IB	TH	NA	NA	NA
	Trecourt et al	2023	37307879	LMO	*KAT6B::KANSL1*	uterine	48	IB	TH	NA	NA	NA
	Trecourt et al	2023	37307879	LGESS	*KAT6B::KANSL1*	uterine, prevesical nodule	70	IIIB	TH-BS, and prevesical nodule resection	yes (38 months)	AWD	38
5	Kommoss et al	2024	this study	KANSL1 sarcoma	*KAT6B::KANSL1*	uterine	51	IA	TH-BSO	no	NED	13
2	Kommoss et al	2024	this study	KANSL1 sarcoma	*KAT6B::KANSL1*	uterine	50	IB	TH-BSO, omentectomy	no	NED	38
14	Kommoss et al	2024	this study	KANSL1 sarcoma	*KAT6B::KANSL1*	uterine	62	IB	NA	no	NED	15
16	Kommoss et al	2024	this study	KANSL1 sarcoma	*KAT6B::KANSL1*	uterine	83	IB	NA	no	NED	8
15	Kommoss et al	2024	this study	KANSL1 sarcoma	*KAT6B::KANSL1*	uterine	54	IB	NA	NA	NA	NA

## Discussion

Herein we report the clinicopathologic and molecular features of a cohort of uterine tumours harbouring the *KAT6B/A::KANSL1* gene fusion. The major novel finding of our study is that *KAT6B/A::KANSL1* uterine tumours are defined by a specific DNA methylation signature that is distinct from other uterine mesenchymal neoplasms, indicating they constitute a distinct type of uterine mesenchymal tumour. These findings are in line with a recent study indicating that *KAT6B::KANSL1* uterine tumours are characterized by a distinct expression profile. In that study, Trecourt et al. demonstrated that, based on unsupervised clustering of RNA expression profiles, *KAT6B::KANSL1* uterine tumours grouped homogeneously and were distinct from ESN/LGESS, HGESS, and uterine smooth muscle tumours [6]. In addition, *KAT6B/A::KANSL1* uterine tumours lack evidence of Wnt/β-catenin pathway activation that is typically seen in LGESS [15]. It is worth noting that while a significant subset of *KAT6B/A::KANSL1* uterine tumours exhibits focal sex cord differentiation, they displayed a methylation profile that was also distinct from UTROSCTs. Given the distinct global DNA methylation and gene expression profile, we advocate for *KAT6B/A::KANSL1* uterine tumours to be recognized as a distinct type of uterine sarcoma—*KAT6B/A::KANSL1* uterine sarcomas.

 Perhaps surprisingly, in our study, *KAT6B/A::KANSL1* uterine sarcomas with high-grade histological features exhibited a variable DNA methylation profile distinct from the core *KAT6B/A::KANSL1* cluster tumours. These outliers clustered near other known genotypes and phenotypes of uterine mesenchymal tumours. Nevertheless, a review of the immunohistochemical profiles of the 5 outliers showed that they did not align with the specific tumor groups which they clustered near. The reason for this divergence between DNA methylation profiles and bona fide tumor differentiation remains unclear. Typically, the DNA methylation profiles of cancer cells reflect the characteristics of their cell of origin. While this could indicate that morphologically high-grade *KAT6B/A::KANSL1* uterine sarcomas originate from different progenitor cells in the uterus, in the light of the divergent immunohistochemical profiles, it is perhaps more likely that high-grade histologic transformation in *KAT6B/A::KANSL1* uterine sarcomas represents a shift in cellular context to either a more primitive cellular state or a transdifferentiated cellular state. This shift may coincide with an altered DNA methylation profile, the presence of a greater degree of CNVs, and genomic instability. Moreover, while uterine sarcomas with recurrent and "simple" genetic alterations, such as gene fusions, are typically considered genomically stable tumours, recent studies of various high-grade, fusion-driven uterine tumours have shown that co-occurring and likely secondary CNVs may occur in a significant number of cases. For example, in *YWHAE::NUTM2A/B* HGESS and inflammatory myofibroblastic tumour, *CDKN2A* deletions are associated with aggressive behaviour [16, 17]. In the current study, *KAT6B/A::KANSL1* uterine sarcomas falling into the core DNA methylation cluster were consistently histologically bland and showed no significant CNVs. In contrast, three outlier cases showed various CNVs, including deletions at the *CDKN2A*/*B* and/or *NF1* loci. This raises the question of whether secondary genomic alterations could play a role in high-grade transformation of *KAT6B/A::KANSL1* uterine sarcomas, potentially correlating with an aggressive clinical course. To address these questions, it is essential to conduct studies on larger cohorts *KAT6B/A::KANSL1* uterine sarcomas with low-grade and high-grade histologic features, particularly cases that contained both components synchronous or metachronously.

To gain a better understanding of the clinical behaviour of *KAT6B/A::KANSL1* uterine sarcoma, we performed a meta-analysis of the cases reported herein, as well as previously published cases with available follow-up. The results highlight that approximately one-third of tumors harboring the *KAT6B/A::KANSL1* gene fusion recur, and some patients ultimately die of disease. It is, however, possible that, as with other rare tumour types, initial series reported may be skewed by tertiary referral centre bias and that the actual clinical behaviour of *KAT6B/A::KANSL1* uterine sarcomas may be less aggressive. Future studies looking at population-based series are needed to provide further insights.

Though preliminary, our findings suggest that histological grading of *KAT6B/A::KANSL1* uterine sarcomas may result in a more meaningful diagnostic classification of these tumours. For instance, tumours showing ESN/LGESS and/or leiomyoma-like features with bland nuclei and low mitotic activity could be classified as low-grade *KAT6B/A::KANSL1* uterine sarcomas, while tumours showing concurrent monomorphic nuclear atypia (large, sometimes epithelioid, nuclei) and elevated mitotic activity (readily identified mitotic figures) could be classified as high-grade *KAT6B/A::KANSL1* uterine sarcomas. Future studies examining an expanded series of clinically annotated *KAT6B/A::KANSL1* uterine sarcomas showing low-grade and high-grade histologic features are needed to evaluate the clinical need for histologic grading. Moreover, the value of molecular studies described herein—including DNA methylation profiling, analysis of the degree of genomic instability, and evaluation of specific secondary genomic alterations, such as *CDKN2A/B* deletions—in classifying *KAT6B/A::KANSL1* uterine sarcoma into clinically relevant groups needs to be studied in larger cohorts.

Given the incomplete current understanding of these neoplasms, we suggest that all uterine mesenchymal neoplasms with overlapping morphology and immunophenotype between endometrial stromal and smooth muscle neoplasms undergo molecular testing to identify a *KAT6B/A::KANSL1* fusion*.* More specifically, we recommend testing for tumors exhibiting the following histologic features, particularly in the context of a “myomectomy-type’ resection: 1) Tumours displaying histologic and immunophenotypic features intermediate between an ESN with classic histology and cellular or highly cellular leiomyoma. This includes tumors resembling ESN but containing scattered thick-walled vessels with perivascular hyalinization throughout, as well as those suspected to be a highly cellular leiomyoma primarily due of the presence of scattered thick-walled vessels. 2) Tumours showing whorled spindle cell proliferations with fibrous to fibromyxoid stroma (fibroblastic/fibromyxoid LGESS-like) and a prominent hemangiopericytomatous vascular network with scattered thick-walled vessels and perivascular hyalinization throughout. 3) Tumours with the aforementioned features that also contain a high-grade round cell component or are suspected to have recurred as a high-grade round cell malignancy. In these scenarios, molecular testing should ideally cover not only the *KAT6B/A::KANSL1* fusion but also other relevant fusions associated with endometrial stromal tumors.

In terms of clinical management, despite their typically bland cytologic features, *KAT6B/A::KANSL1* uterine sarcomas ideally require at least total hysterectomy and ideally bilateral salpingo-oophorectomy also in ER-positive cases for definitive surgical management. “Myomectomy-type” resection does not appear to be sufficient as the tumour can recur locally [5, 6]. When exhibiting malignant cytologic features either in the primary or recurrent tumours, *KAT6B/A::KANSL1* uterine sarcomas can pursue a rapidly progressive clinical course, and there is currently no known effective systemic therapy (chemotherapy or targeted therapy) for this tumour type.

In conclusion, *KAT6B/A::KANSL1* uterine sarcoma is a molecularly unique tumour that should be recognized as a distinct entity. While most tumours display low-grade histologic features, a subset have high-grade histologic features that is accompanied by a divergent methylation profile and a higher number of CNVs, which appears to correlate with a more aggressive clinical trajectory.

## Supplementary information

Below is the link to the electronic supplementary material.

Supplementary file1 (XLSX 10 KB)

## Data Availability

The data for this study data are available upon reasonable request.
